# Compliance of functional exercises in school-age children with limb fractures: implication for nursing countermeasures

**DOI:** 10.1186/s12887-022-03193-6

**Published:** 2022-03-14

**Authors:** Hui Liu, Yun Wang, Mengya Li, Dan Chen, Yuping Tang

**Affiliations:** 1 Department of Nursing, Children’s Hospital of Nanjing Medical University, Nanjing, China; 2 Gulou District, No. 72, Guangzhou Road, Hunan Road Street, Nanjing City, Jiangsu Province, China

**Keywords:** Compliance, Functional exercises, Children, Fractures, Care, Nursing

## Abstract

**Background:**

Functional exercises is very essential to the recovery of patients with fracture. We aimed to evaluate the compliance of functional exercises in school-age children with limb fracture, to provide evidence to the clinical management and nursing care of children with limb fracture.

**Methods:**

School-age children with limb fractures treated in our hospital from January 1, 2020 to June 30, 2021 were selected. The characteristics and postoperative functional exercise compliance of included children were analyzed. Pearson correlation and Logistic regression analysis were conducted to analyze the influencing factors of compliance to functional exercises.

**Results:**

A total of 328 children with limb fracture were included, the incidence of compliance to functional exercise was only 35.98%. Pearson correlation analysis showed that age(*r* = 0.707), only child of family(*r* = 0.537), guardians(*r* = 0.642) and type of temperament(*r* = 0.635) were correlated with compliance to functional exercises in school-age children with limb fractures (all *p* < 0.05). Logistic regression analysis indicated that age ≤ 10y (OR2.913, 95%CI2.091 ~ 3.611), only child of family (OR2.006, 95%CI1.683 ~ 2.558), guarded by grandparents (OR1.512, 95%CI1.201 ~ 2.118), non-easy-going temperament (OR4.127, 95%CI3.811 ~ 4.902) were the influencing factors of non-compliance to functional exercises in children with limb fracture (all *p* < 0.05).

**Conclusions:**

School-age children have poor compliance with functional exercises after limb fractures, and there are many influencing factors. For children with those risks, health care providers should actively intervene in nursing to improve children’s exercise compliance and the rehabilitation effect.

## Background

Previous studies [1–3] have reported that fractures in school-age children are very common in clinical practice, with an incidence ranging from 2.04% to 11.46%. School-age children are in the stage of gradual bone ossification [4]. The bone composition contains more colloids, less calcium, and full of elasticity, but they are still not strong and are prone to fractures [5]. Fractures of the limbs are more common in school-age children, mainly due to the higher physical and psychological maturity of children during this period, and heavier curiosity about things, less self-protection ability and life experience [6, 7]. Besides, those children spend a relatively long time in school during this period, and parents’ awareness of protection is relatively relaxed, resulting in children are prone to fractures during daily activities [8, 9]. Therefore, the prevention and care of school-age children's fractures have an important influence on the prognosis of children.

School-age children are the most vigorous period of the development of various physiological functions in their lives [10]. There is a big difference in the anatomy, injury mechanism and healing characteristics of adults [11]. Fracture injuries in children are often accompanied by bone damage. Improper treatment will lead to shorter bone development and deformities such as crooked, oblique, etc. [12, 13]. Children have a high disability rate and are accompanied by varying degrees of dysfunction, but their healing speed is faster than that of adults [14]. Therefore, how to promote the functional recovery of children with fractures is particularly important. With the development and improvement of medical technology, people have higher requirements for the treatment of diseases. They are not only satisfied with curing the disease and injury itself, but also pay more attention to the long-term prognosis of the disease. Therefore, in the process of fracture treatment, the functional exercise of the affected limb is particularly important, which directly has a serious impact on the later functional recovery [15]. The latest rehabilitation concept proposes that in the acute phase, symptoms including congestion, swelling, increase in the number of fibers and collagen cells, and shedding of necrotic cells at the acute stage will cause local adhesions, which will affect the later functional exercises [16, 17]. Effective functional exercises in the early stage of the fracture will promote local blood circulation, reduce swelling, prevent compartment syndrome, and promote functional recovery [18]. Previous studies [19, 20] have shown that children with early fractures suffer from severe pain, coupled with the unfamiliarity of the environment during hospitalization, which makes them feel nervous, scared, and crying, resulting in poor exercise compliance, missing the best exercise opportunity, and directly affecting the effect of surgery and quality of life of children in later stages. Therefore, it is necessary to explore the factors affecting the compliance of functional exercises in school-age children with limb fracture, to provide evidence support for clinical treatment and care of children with limb fracture.

## Methods

### Ethics

In this study, all methods were performed in accordance with the relevant guidelines and regulations. This present study was a prospective cohort study design, the study had been checked and approved by the ethical committee of our hospital with approval number: (202001011–2). Furthermore, the written informed consents had all been obtained from the guardians of included children, and children’s assent to participate in the study was obtained accordingly.

### Population

This study selected school-age children with limb fractures who admitted to our department from January 1, 2020 to June 30, 2021 as the research population. The inclusion criteria for children were as following: ①The children were school-age children aged 6 to 15 years old; ②The children could understand language expressions; ③The children could express their ideas and thought on their own; ④The children have accompanied guardians during their hospitalization; ⑤The guardian of the child was aware of the purpose of this study and voluntarily signed the informed consent form. Exclusion criteria for children were as following: ①Children with old fractures; ②Children with congenital diseases, such as congenital heart disease, congenital bone malformations; ③Children with visceral damage, such as liver rupture, spleen rupture, etc.; ④Children or their guardians were unwilling to participate in this study.

### Definition and evaluation of functional exercise compliance

Exercise compliance was defined as the degree of consistent behavior of children performing rehabilitation exercises according to the requirements and recommendations of medical staff. We used the previously reported postoperative functional exercise compliance questionnaire [21] to analyze the children’s exercise compliance. The questionnaire involved postoperative functional exercise knowledge, doctor’s execution, daily functional exercise time, daily functional exercise times, conscious exercise and daily life behavior ability. Each item used a 5-level scoring method according to the content of the answer, corresponding to 0 ~ 4 points, the higher the score indicated the better the compliance, and scores of 3 points or above of all items indicated full compliance, otherwise it was rated as non-compliance.

### Data collection

Two investigators collected following data from the communication to the guardians or children and related medical records: age, gender, body mass index(BMI), whether the child was the only child of family, place of residence, guardians, type of fracture, site of fracture, causes of fracture and type of temperament. Any disagreement was solved by further discussions.

This study used the Chinese School-age Children Temperament scale (CSTS) [22] to assess the temperament characteristics of children. The questionnaire has a total of 99 items belonging to 9 temperament dimensions, and each item is scored 1 to 6 points in 6 levels: almost never, very rare, rare, common, very common, and almost always. According to the score, the children were rated as easy-going, troublesome, initiate slow and intermediate temperament accordingly. Professionally trained nursing staff used CSTS communicating with family members or guardians who know the children’s life habits best. Family members who understand the children’s life habits have the longest contact time with the children, and observe the children’s behavior for a long and careful time, and fully understand the personality characteristics of children. The nursing staff explained the purpose of the investigation to the family members, and after obtaining their understanding and consent, we correctly guided the guardians to fill in.

### Statistical analysis

In this study, SPSS 23.0 statistical software was used to process the data. The count data were expressed as percentage (%), the comparison between groups was compared by chi-square test, the continuous variables were expressed as mean ± standard deviation, and the comparison between groups was conducted by t test. We selected the statistically significant outcome variables of univariate analysis as independent variables, and took the factors that affect children's functional exercises as dependent variables, and analyzed the influencing factors of children's functional exercises by Logistic regression analysis. Besides, Pearson correlation analysis was conducted to evaluate the compliance to functional exercises and related characteristics. In this study, *P* < 0.05 was considered as the difference between the groups was statistically significant.

## Results

### The characteristics of included children

A total of 328 children with limb fracture were included, of whom 118 children had good compliance to functional exercise, the incidence of compliance to functional exercise in school-age children with limb fractures was 35.98%. As presented in Table 1, there were significant differences in the age, only child of family, guardians and type of temperament between compliance group and non-compliance group (all *p* < 0.05). No significant differences in the gender, BMI, place of residence, type of fracture, site of fracture and causes of fracture between compliance group and non-compliance group were found (all *p* > 0.05).

**Table 1:** The characteristics of included children

Variables	Compliance group(*n* = 118)	Non-compliance group(*n* = 210)	t/χ^2^	p
Age(y)	11.02 ± 2.35	8.96 ± 2.71	3.021	0.005
Male/female	75/43	142/68	2.408	0.091
BMI(kg/m^2^)	21.34 ± 2.44	22.01 ± 3.52	6.663	0.076
Only child of family			1.418	0.002
Yes	36(30.51%)	151(71.90%)		
No	82(69.49%)	59(28.10%)		
Place of residence			2.112	0.107
Rural area	47(39.83%)	85(40.48%)		
City	71(60.17%)	125(59.52%)		
Guardians			5.291	0.013
Parents	79(66.95%)	82(39.05%)		
Grandparents	30(25.42%)	124(59.05%)		
Others	9(7.63%)	4(1.90%)		
Type of fracture			4.119	0.055
Closed	85(72.03%)	149(70.95%)		
Open	33(27.97%)	61(29.05%)		
Site of fracture			2.814	0.054
Upper limb	72(61.02%)	114(54.29%)		
Lower limb	38(32.20%)	75(35.71%)		
Both	8(6.78%)	21(10%)		
Causes of fracture			1.947	0.106
Traffic accident	61(51.69%)	104(49.52%)		
Fall	55(46.62%)	102(48.57%)		
Others	2(1.69%)	4(%)		
Type of Temperament			2.988	0.001
Easy-going temperament	74(62.72%)	60(28.57%)		
Troublesome temperament	17(14.41%)	67(31.90%)		
Initiate slow temperament	16(13.56%)	51(24.29%)		
Intermediate temperament	11(9.32%)	32(15.24%)		

### Pearson correlation analysis

As indicated in Table 2, Pearson correlation analysis showed that age(*r* = 0.707), only child of family(*r* = 0.537), guardians(*r* = 0.642) and type of temperament(*r* = 0.635) were correlated with compliance to functional exercises in school-age children with limb fractures (all *p* < 0.05).

**Table 2:** Pearson correlation analysis of compliance to functional exercises and related characteristics

Variables	r	p
Age(y)	0.707	0.042
Gender	0.214	0.085
BMI(kg/m^2^)	0.109	0.079
Only child of family	0.537	0.044
Place of residence	0.115	0.103
Guardians	0.642	0.007
Type of fracture	0.126	0.073
Site of fracture	0.108	0.115
Causes of fractures	0.205	0.128
Type of temperament	0.635	0.028

### Logistic regression analysis

The variable assignments of multivariate logistic regression were showed in Table 3. As indicated in Table 4, Logistic regression analysis indicated that age ≤ 10y (OR2.913, 95%CI2.091 ~ 3.611), only child of family(OR2.006, 95%CI1.683 ~ 2.558), guarded by grandparents(OR1.512, 95%CI1.201 ~ 2.118), non-easy-going temperament(OR4.127, 95%CI3.811 ~ 4.902) were the influencing factors of non-compliance to functional exercises in children with limb fracture (all *p* < 0.05).

**Table 3:** The variable assignments of multivariate logistic regression

Factors	Variables	Assignment
Non-compliance	Y	Yes = 1, no = 2
Age(y)	X_1_	≤ 10 = 1, > 10 = 2
Only child of family	X_2_	Yes = 1, no = 2
Guardians	X_3_	Grandparents = 1, parents = 2, others = 3
Type of temperament	X_4_	Non-easy-going temperament = 1, easy-going temperament = 2

**Table 4:** Logistic regression analysis on the influencing factors of non-compliance to functional exercises in children with limb fracture

Variables	β	Wald	OR	95%CI	p
Age ≤ 10y	0.115	0.191	2.913	2.091 ~ 3.611	0.015
Only child of family	0.121	0.174	2.006	1.683 ~ 2.558	0.031
Guarded by grandparents	0.148	0.192	1.512	1.201 ~ 2.118	0.024
Non-easy-going temperament	0.141	0.124	4.127	3.811 ~ 4.902	0.008

## Discussions

Early functional exercises for fractures are mostly within 2 weeks after surgery, mainly to exercise muscle contraction and relaxation, and mid-term functional exercises are mostly within 3 to 6 weeks after surgery [23]. The exercise intensity can be gradually strengthened and large joint activities can be attempted, but activities that are not conducive to fracture connection and stability still need to be restricted [24, 25]. Later functional exercises are mostly carried out after 6 weeks after surgery, and the limb function is gradually restored through comprehensive joint and muscle exercises [26]. It is very important to actively carry out nutritional supplements and scientific functional exercises after limb fractures. Postoperative functional exercises can not only avoid complications such as postoperative joint stiffness and muscle atrophy, but also promote the healing of the patient’s fractures [27]. However, functional exercise after surgery is more challenging in school-age children since they are lively and active, and their safety awareness and self-control ability are not strong enough [28, 29]. Currently, the status and influencing factors of compliance to functional exercise in school-age children with limb fractures remain unclear. The results of this study have found that the incidence of compliance to functional exercise in school-age children with limb fractures is 35.98%, and for children with age ≤ 10y, only child of family, guarded by grandparents, non-easy-going temperament, they may have less compliance to functional exercises after surgery, early targeted nursing care are needed for those children to improve the compliance to functional exercises of children.

Functional exercise after limb fracture operation needs to last a relatively long period of time, so functional exercise compliance is a key factor. The results of this study show that school-age children’s compliance with functional exercises after limb fractures is low, which suggests that compliance with functional exercises after school-age children’s limb fractures is affected by multiple factors. Individualized interventions can effectively improve the compliance of school-age children with limb fractures [30]. Functional exercise compliance helps children recover as soon as possible. Compliance refers to the extent to which patients accept and obey the prescribed medical and nursing measures and their behaviors [31]. This study has investigated and analyzed the compliance of school-age children with limb fractures, and has found that children under 10 years of age have poor early exercise compliance, mainly due to their relatively young age, relatively poor understanding and communication skills, and self-care ability, the importance of functional exercise cannot be fully understood for those children. Therefore, for children with age ≤ 10y, the health care providers should use simple and understandable language to communicate with them when they perform functional exercises, and integrate the exercise with games to increase the children’s interest in exercise, and then improve their compliance to functional exercise.

Children with limb fractures will still have obvious pain, swelling and movement dysfunction in the early postoperative period. The children will be disturbed by physical discomfort or emotional state of tension and fear [32, 33]. The older the school-age children can be more adapt well to the postoperative state of fractures and better cooperate with functional exercises. Children whose only children and their main caregivers are grandparents receive more attention in their daily lives, they have relatively poor tolerance to pain [34]. When they cannot maintain functional exercises, their caregivers are more likely to compromise, give up, thereby reducing the compliance [35]. Temperament characteristics are the psychological characteristics of a person's personality, and it is reported that it interacts with physical diseases and the treatment of physical diseases [36, 37]. School-age children with easy-going temperament have a weaker stress response to fractures and surgical treatments, they have strong receptivity, positive emotions, and compliance with functional exercises [38]. Therefore, in the functional exercise work of school-age children after limb fracture surgery, it is necessary to integrate the above influencing factors and pay attention to the relevant characteristics of individuals. The fun of functional exercise can be improved through methods such as children's game-style functional exercise and goal completion reward programs to help children improve their compliance.

Studies [39] have shown that the complexity of the treatment plan may influence the patients’ compliance. Although school-age children have developed cognitive abilities, they have poor understanding of things and self-control, things and treatment programs cannot be understood easily [40]. Therefore, when formulating early functional exercise programs for children with affected limbs, attention should be paid to explanations as simple as possible, focus on demonstrations, and proceed in a planned and segmented manner, so that the children can easily accept and understand. The general exercise method is from simple to complex, time from short to long, frequency from less to more, the intensity of exercise gradually increases, and the transition from passive exercise to active exercise gradually [41, 42]. It is appropriate for the child to accept and not cause severe pain, and if necessary, give appropriate analgesics in accordance with the doctor's instructions before the exercise [43, 44], so that the children can gradually adapt and not have fear of the pain caused by the functional exercise of the affected limb, and increase their compliance with the early functional exercise.

Several limitations in this present study must be considered. Firstly, participants with ≥ 12 years should be considered as adolescents in a separate group since there are important differences between these two groups that affect compliance, however, in China age ≥ 16 years is generally considered as adolescents, most of our included children are in the age range of 9–12 years old, it may underpower to detect the group between this two group. Secondly, our study is a single centered observational study, the sample size is small, and there may some other variables that affect postoperative functional exercises. Therefore, multi-centered studies with larger sample size and rigorous design in different areas are needed to further evaluate the children’s compliance with postoperative functional exercises in the future.

## Conclusions

In summary, school-age children’s compliance with postoperative functional exercises for limb fractures is low, which should arouse great attentions from health care providers and related guardians. Compliance with postoperative functional exercises is affected by many factors including the age of the child, the only child, the main caregiver, and the characteristics of temperament. Those influencing factors should be considered to take targeted intervention measures to help children and their parents correctly and actively carry out postoperative functional exercises, to improve the compliance with postoperative functional exercises, thereby improving the recovery of children.

## Data Availability

All data generated or analyzed during this study are included in this published article.

## Abbreviations

BMI: Body mass index
CTST: Chinese School-age Children Temperament scale
